# Kidney function improvement after urinary diversion for cisplatin eligibility in bladder cancer patients

**DOI:** 10.14440/bladder.2024.0034

**Published:** 2025-03-03

**Authors:** Fernando Korkes, José Henrique DallAcqua Santiago, André Marantes Masciarelli Pinto, Artur da Silva Farias, Frederico Timóteo, Suelen Patricia dos Santos Martins, Sidney Glina

**Affiliations:** Division of Urology, Centro Universitario FMABC, Santo Andre, São Paulo 09060-650, Brazil

**Keywords:** Urothelial cancer, Bladder neoplasm, Nephrostomy, Ureteral stent, Urinary diversion, Renal insufficiency, Cisplatin, Neoadjuvant chemotherapy

## Abstract

**Background:**

Muscle-invasive bladder cancer (MIBC) is an aggressive disease typically treated with radical cystectomy following neoadjuvant chemotherapy (NAC). However, the presence of hydronephrosis – a significant marker of advanced disease – can impair renal function, potentially precluding patients from receiving cisplatin-based NAC.

**Objective:**

The present study aimed to assess the role of urinary diversion in patients with MIBC, specifically in reversing renal function impairment and enabling eligibility for cisplatin-based therapy.

**Methods:**

A retrospective study was performed by evaluating a database of patients treated for urothelial MIBC from 2018 to 2021. Case notes were reviewed to identify patients with hydronephrosis who underwent urinary diversion. The types of urinary diversion recorded included percutaneous nephrostomy, ureteral stenting, or surgical obstruction release. Renal function was assessed retrospectively using the glomerular filtration rate (GFR), estimated from creatinine clearance.

**Results:**

Records of a total of 72 patients were evaluated. The mean GFR before urinary diversion was 44.1 ± 26.4 mL/min, which improved to 59.1 ± 31.9 mL/min post-diversion, resulting in a mean GFR improvement of 15.0 ± 20.0 mL/min. Forty-four patients had an initial GFR below 50 mL/min, with 75% of them achieving a GFR >50 mL/min after urinary diversion. More than half of these patients (*n* = 25, 56%) experienced an improvement to a GFR exceeding 60 mL/min. The time to reach the best GFR varied widely (mean: 59 ± 33 days, range 9 – 165 days). Logistic regression analysis identified initial GFR as a significant predictor of GFR recovery (odds ratio = 1.11, 95% confidence interval = 1.02 – 1.21, *p* = 0.012).

**Conclusion:**

Urinary diversion can benefit patients with upper tract obstruction secondary to MIBC by improving renal function, thereby enabling eligibility for cisplatin-based chemotherapy. Notably, the time to GFR recovery following urinary diversion varied among individuals.

## 1. Introduction

Muscle-invasive bladder cancer (MIBC) is an aggressive disease associated with elevated mortality rates. Radical cystectomy (RC) remains the cornerstone of treatment for these patients. There is now substantial evidence supporting the use of cisplatin-based systemic therapy before surgery for all eligible patients.^1^ In this context, neoadjuvant chemotherapy (NAC) has been shown to reduce the risk of mortality by up to 16% and may improve 5-year survival by up to 8%.^2^-^4^

Despite the body of evidence supporting NAC, these data have not been widely translated into the routine implementation of multimodal strategies for treating MIBC. Factors, such as increasing age, comorbidities, lower patient income, and treatment at non-academic centers have been identified as barriers to the acceptance of NAC.^5^ Concerns regarding the toxicity of chemotherapy, delays in the time to cystectomy, and the potential overtreatment of patients with organ-confined disease further contribute to its underuse. In addition, frail patients with MIBC may have clinical characteristics that contraindicate the use of cisplatin-based chemotherapy regimens.^6^

Contraindications to cisplatin-based chemotherapy include hearing loss/dysfunction, cardiac dysfunction, poor performance status, and renal insufficiency.^7^,^8^ A significant proportion of MIBC patients have impaired renal function, and when taking a creatinine clearance (CrCl) below 60 mL/min as the threshold, studies have demonstrated that approximately 40% of MIBC patients over the age of 70 may be ineligible for cisplatin-based chemotherapy.^9^ Multiple factors contribute to renal function impairment in this patient population, including comorbidities, age-related declines in glomerular filtration rate (GFR), and ureteral obstruction. It is important to note that one major limitation of CrCl testing is its decreasing accuracy as GFR declines, due to the increasing tubular secretion of creatinine. As a result, GFR may be overestimated in patients with renal impairment.

The incidence of ureteral obstruction and hydronephrosis in MIBC ranges from 7.2% to 54.1%.^10^-^12^ This condition typically arises from intramural or extravesical tumoral extension, tumor involvement of the ureteral orifice, or simultaneous ureteral tumors.^13^ The negative impact of ureteral obstruction in this cohort is multifaceted. Hydronephrosis serves as a significant marker for advanced disease and is an independent prognostic marker for adverse oncological outcomes, such as recurrence-free survival and cancer-specific survival.^10^ Moreover, urethral obstruction can result in renal function impairment, which may prevent patients from benefiting fully from cisplatin-based NAC.

Accurately predicting the potential for kidney function recovery after the release of urinary obstruction is crucial for both urologists and oncologists. Functional recovery can occur as early as 7 – 10 days, though it may take longer, depending on factors, such as the completeness and duration of the obstruction, as well as the function of the contralateral kidney.^14^,^15^

There is a lack of studies evaluating the impact of urinary obstruction release on kidney function, specifically in the MIBC context. The present study aimed to assess the role of urinary diversion in patients with urothelial MIBC, particularly its effect on reversing renal function impairment, and to discuss the various factors that may influence the recovery of GFR to levels meeting cisplatin eligibility.

## 2. Methods

### 2.1. Patients

A retrospective study was conducted to examine the patients treated for urothelial MIBC at our institution from 2018 to 2021. All case notes were reviewed, and patients with hydronephrosis who had undergone urinary diversion were included for further analysis. The types of urinary diversion recorded included placement of a percutaneous nephrostomy (PCN), insertion of a ureteral stent, transurethral resection of the bladder tumor (TURBT) over the ureteral orifice, or surgical obstruction release (including cutaneous ureterostomy alone or incontinent urinary diversion). The decision regarding the method of diversion was made at the discretion of the surgeon.

Data collected for analysis included demographic information, serum creatinine levels, and post-operative complications. Patients in the surgical group who underwent orthotopic diversions were excluded from further analysis. Serum creatinine was measured using kinetic colorimetric assays. GFR was determined based on CrCl, calculated from serum creatinine using the Cockcroft-Gault (CG) formula.^16^ We selected the CG formula as the primary tool for estimating CrCl, as it has similar efficiency to the Chronic Kidney Disease Epidemiology Collaboration equation.^17^

### 2.2. Statistical analysis

Statistical analysis was performed using STATA 14.0 (StataCorp LP, USA). Groups were compared using Pearson’s Chi-square or Fisher’s exact test. The Student’s *t*-test was applied for continuous variables with a normal distribution, while the Mann–Whitney U test was used for variables with a non-normal distribution. Analysis of variance was performed for multiple comparisons. A generalized linear regression model was utilized to investigate the effect of covariates on the GFR recovery. Statistical significance was defined as a *p* < 0.05.

### 2.3. Ethical approval and patient consent

All participants voluntarily provided informed consent and were aware that they could withdraw consent at any time. The study was approved by the Institutional Review Board (IRB), with approval numbers of 40836920.0.0000.0071 and 26817719.2.0000.0082.

## 3. Results

A total of 72 patients satisfied the criteria for having hydronephrosis secondary to MIBC that was subsequently relieved. Of these patients, 51 were male and 21 were female. The mean age of the study population was 67.5 years (range, 33 – 92 years). Demographic data are presented in Table 1.

**Table 1:** Study population demographics

Parameter	*n*/mean	%/range
Gender		
Male	51	71.0
Female	21	29.0
Ethnicity		
White	52	72.2
Black	13	18.1
Brown	7	9.7
Hydronephrosis		
Unilateral	41	56.9
Bilateral	29	40.3
Hydronephrosis right		
Absent	25	34.7
Mild	13	18.1
Moderate	17	23.6
Severe	17	23.6
Hydronephrosis left		
Absent	19	26.4
Mild	9	12.5
Moderate	30	41.7
Severe	14	19.4
Pre-creatinine	2.4	(0.5 – 12.6)
Pre-creatinine clearance	44.1	(5.1 – 113.0)
Post-creatinine	1.4	(0.5 – 4.2)
Post-creatinine clearance	59.1	(9.5 – 165.3)
Urinary diversion		
Unilateral	11	15.3
Bilateral	61	84.7
Right kidney diversion		
Ureteral stent	5	6.9
Nephrostomy tube	4	5.6
Surgical diversion	18	25.0
TURBT	25	34.7
Left kidney diversion		
Ureteral stent	4	5.6
Nephrostomy tube	5	6.9
Surgical diversion	15	20.8
TURBT	27	37.5

Abbreviation: TURBT: Transurethral resection of the bladder tumor.

**Figure 1:**
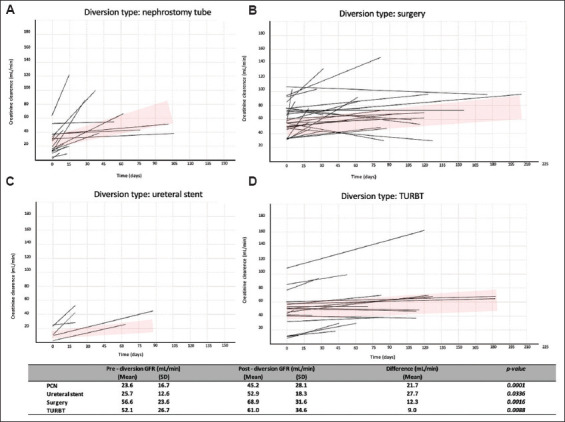
Recovery of creatinine clearance following PCN (A), surgery (B), ureteral stent placement (C), and TURBT (D) Abbreviations: PCN: Percutaneous nephrostomy; TURBT: Transurethral resection of the bladder tumor.

**Figure 2:**
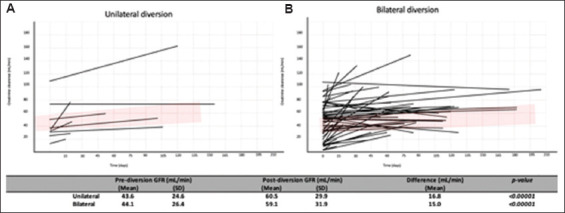
Recovery of creatinine clearance following unilateral (A) and bilateral (B) diversion

The mean GFR before urinary diversion was 44.1 ± 26.4 mL/min (range, 5.1 – 113 mL/min), while the mean GFR after urinary diversion was 59.1 ± 31.9 mL/min (range, 9.5 – 165.3 mL/min). After urinary diversion, the mean GFR recovery was 15.0 ± 20.0 mL/min (range, 0 – 73.6 mL/min). The mean GFR recovery for different types of urinary diversion was as follows: PCN, 22 mL/min (range, 0 – 74 mL/min); ureteral stent, 27 mL/min (range, 0 – 42 mL/min); surgery, 12 mL/min (range, 0 – 58 mL/min); and TURBT, 9 mL/min (range, 0 – 52 mL/min). GFR was significantly higher after all types of diversion (Figure 1), as well as after unilateral and bilateral diversion (Figure 2).

**Figure 3:**
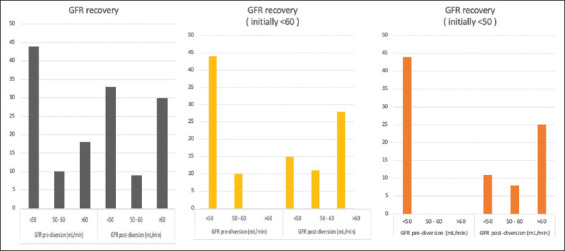
Recovery of glomerular filtration rate according to pre-diversion values

Of the 72 patients, 44 had an initial GFR below 50 mL/min. 75% of these patients (*n* = 33) demonstrated an improvement in GFR to above 50 mL/min following urinary diversion. More than half of these patients (*n* = 25, 56%) experienced an increase in their GFR to above 60 mL/min (Figure 3). In addition, 54 patients had an initial GFR below 60 mL/min, and 51.8% (*n* = 28) of these patients experienced an improvement in GFR (Figure 3).

**Figure 4:**
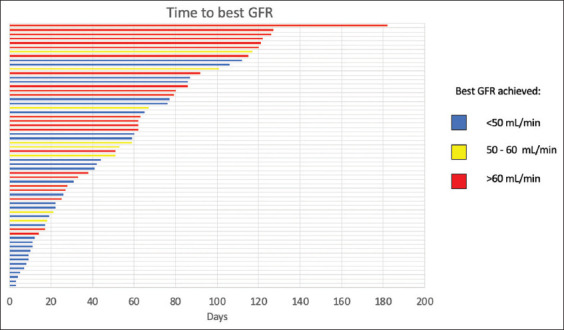
Time to achieve the best glomerular filtration rate

The time to achieve the best GFR varied widely. The median ± standard deviation (SD) time to reach the optimal GFR was 59 ± 33 days (range, 9 – 165 days). Thirteen patients with a baseline GFR below 50 mL/min (mean GFR ± SD = 36.8 ± 12.7 mL/min) achieved a GFR above 50 mL/min after a mean period of 61 ± 39 days (mean GFR ± SD = 70.0 ± 15.4 mL/min). When using a cutoff of GFR below 60 mL/min, 16 patients showed a significant recovery. These patients initially had a mean GFR of 46.8 ± 15.9 mL/min, and after a mean period of 78.3 ± 42 days, their GFR increased to 76.6 ± 15.7 mL/min (Figure 4).

Eleven patients could be classified as “super-recoverers.” These patients had a very low initial GFR (mean GFR ± SD = 38.5 ± 20.2 mL/min), and after a relatively short period (29 ± 18 days), their GFR increased to 82 ± 25 mL/min. Most of these patients had mild- to high-grade hydronephrosis in their renal units (13/12 units, 60%), and nearly all underwent bilateral urinary diversion (10/11, 91.1%).

**Figure 5:**
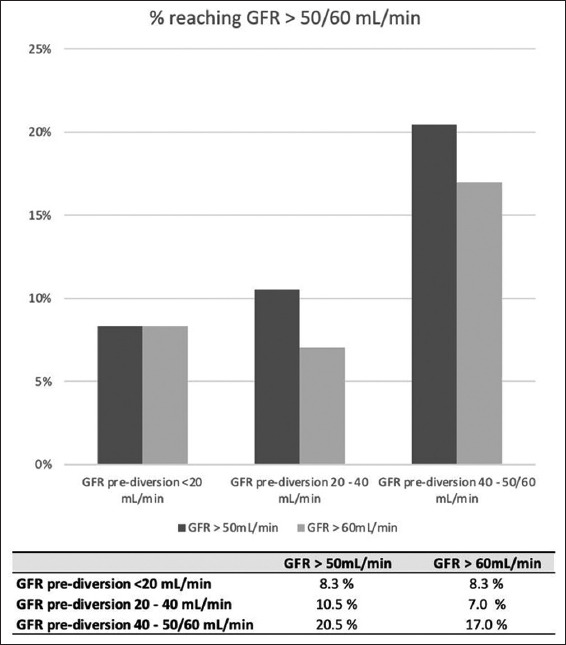
Recovery of glomerular filtration rate according to pre-diversion glomerular filtration rate categories

Logistic regression indicated that the initial GFR was significantly associated with GFR recovery (odds ratio = 1.11, 95% confidence interval = 1.02 – 1.21, *p* = 0.012). For each unit increase in pre-diversion GFR, the odds of a patient becoming cisplatin-eligible increased by 11.32 (Table 2). Figure 5 summarizes the percentage of patients achieving a ClCr >50 mL/min and >60 mL/min based on initial GFR categories.

**Table 2:** Multivariate logistic regression for glomerular filtration rate recovery

Parameter	Multivariate regression	

	Odds ratio (95% confidence interval)	*p*-value
Hydronephrosis		
Unilateral	Reference	-
Bilateral	1.22 (0.22 – 6.89)	0.822
Hydronephrosis grade		
Mild	Reference	-
Moderate	8.75 (0.46 – 168.02)	0.150
Severe	9.56 (0.45 – 202.69)	0.147
GFR pre	1.11 (1.02 – 1.21)	0.012*
GFR cluster		
<50 mL/min	Reference	-
50 – 60 mL/min	4.42 (0.20 – 97.66)	0.346
>60 mL/min	2.10 (0.03 – 133.08)	0.726
Urinary diversion		
Unilateral	Reference	-
Bilateral	3.42 (0.22 – 52.92)	0.379
Diversion type		
Ureteral stent	Reference	-
PCN	0.42 (0.03 – 5.56)	0.511
Surgery	0.06 (0.01 – 2.04)	0.119
TURBT	0.05 (0.01 – 1.33)	0.073

Note:

* indicates statistical significance (*p*<0.05). Abbreviations: CI: Confidence interval; GFR: Glomerular filtration rate; PCN: Percutaneous nephrostomy; TURBT: Transurethral resection of the bladder tumor.

## 4. Discussion

MIBC is a challenging disease. NAC with cisplatin-based combination regimens lowers the risk of recurrence following RC.^18^ As mentioned earlier, several factors can determine cisplatin ineligibility, with a ClCr <60 mL/min being one of the primary conditions that preclude MIBC patients from benefiting from NAC. Virtually, half of the patients with MIBC are ineligible for NAC, and hydronephrosis is a significant factor associated with reduced GFR.^8^

Despite concerns about NAC for MIBC, such as therapeutic toxicity and potential delays to RC, robust scientific evidence indicates that the benefits of NAC outweigh the risks for eligible patients.^1^-^4^ It is essential to emphasize that urothelial carcinoma of the bladder is highly chemosensitive and generally responds well to cisplatin-based regimens.^1^,^19^ Occult metastasis at the time of diagnosis is a key reason for the poor prognosis of MIBC, with recurrence rates reaching as high as 50% after RC.^18^,^20^ The rationale for administering NAC is to increase survival by targeting micrometastatic disease at the time of diagnosis when the tumor burden is at its lowest.^21^

A common dilemma in the management of MIBC is whether to perform upfront RC or a urinary diversion followed by NAC. RC is a cornerstone of curative treatment for MIBC and should not be delayed for more than 12 weeks, as postponing the procedure increases mortality risk.^22^ Thus, it is crucial to ensure that NAC does not interfere with the patient’s ability to undergo surgery. While RC is a complex procedure with a high 90-day complication rate, it can lead to the inability to administer adequate post-operative chemotherapy in over 30% of patients.^23^ This scenario highlights the complexity of decision-making, where urologists and clinical oncologists must carefully consider whether performing a urinary diversion to improve renal function may enable cisplatin-ineligible patients to benefit from NAC. Therefore, appropriate patient selection for urinary diversion is of utmost importance.

Since hydronephrosis is a marker of both advanced disease and poor prognosis, it is reasonable to assume that MIBC patients with renal obstruction may benefit from the advantages of NAC. Ureteral obstruction elevates ipsilateral ureteral pressure and reduces renal blood supply, leading to a decrease in GFR and cellular and molecular abnormalities in the obstructed kidney, ultimately progressing to fibrosis.^24^ Upon relief of the obstruction, the renal response depends on several factors, including the patient’s age, ureteral compliance, duration of the obstruction, contralateral kidney function during the obstruction, and the degree of fibrosis.^24^ Notably, the duration of ureteral obstruction plays a critical role in the kidney’s regenerative potential.

The recovery period following the release of an obstruction has been widely studied in animal models.^25^ In rats, functional and structural recovery of a kidney obstructed for 3 – 7 days typically takes 4 – 6 weeks.^25^,^26^ In dogs, after 4 days of obstruction, there is a practically complete recovery of the GFR. However, as the obstruction persists, the recovery potential progressively declines, such that after 21 days, only 13% of the original GFR is restored.^27^ In humans, the exact recovery timeline is less defined. While the literature generally agrees that complete GFR recovery is possible when diversion is implemented promptly, controversy remains regarding the recovery potential for kidneys obstructed for longer periods.^15^,^28^-^32^ This uncertainty may stem from the difficulty in accurately assessing the degree of obstruction in humans, as well as challenges in estimating the duration of obstruction in patients diagnosed with bladder cancer.

In our own observations, there was considerable variability in recovery time among patients (Figure 4). While some patients demonstrated GFR recovery within a few days, others required several weeks for full recovery. Given that MIBC treatment is time-sensitive, the time required for GFR recovery is vital, as adequate GFR is necessary for administering NAC and proceeding with surgery. The overall median time to achieve the best GFR was 59 days, which can be considered a relatively long period in the context of this time-sensitive disease. However, we identified 11 patients with very low initial GFR (mean ± SD = 38.5 ± 20.2 mL/min) who attained a GFR of 82 ± 25 mL/min after a relatively short period following the release of obstruction. Approximately 60% of these “super-recoverers” had moderate to severe hydronephrosis, and 91.1% had undergone bilateral diversion.

To identify patients more likely to achieve GFR recovery, we performed a multivariate analysis, which revealed that the only statistically significant predictor of GFR recovery was the initial GFR (Table 2). A previous prospective study of non-oncological patients with ureteral obstruction found that several factors, such as pre-operative renographic GFR, renal perfusion, parenchymal thickness, parenchymal echogenicity, corticomedullary differentiation, reduction of the renal resistive index, and compensatory hypertrophy of the contralateral normal kidney, were associated with renal function recovery after the relief of obstruction. However, on multivariate analysis, only pre-operative GFR and renal perfusion remained statistically significant as independent factors affecting kidney functional recovery.^14^ Our data corroborate these findings, demonstrating that pre-operative GFR was the sole independent factor influencing renal function recovery in patients with bladder cancer and hydronephrosis. Patients with a GFR between 40 and 50 mL/min had the highest likelihood of becoming eligible for cisplatin. In contrast, kidneys with a GFR of around 10 mL/min were likely irreversibly damaged, as shown in Figure 5. Therefore, patients with such low GFR should not be considered for diversion as a strategy to improve their chances of receiving cisplatin (Figure 5).

Although we could not identify significant predictors for the time to GFR recovery, we have demonstrated that some patients can become eligible for cisplatin, and this can occur within a reasonable timeframe. Future studies that evaluate imaging techniques and attempt to establish GFR recovery nomograms may provide valuable insights. In the meantime, it is essential to note that 75% of patients who were initially ineligible for cisplatin became eligible after upper tract deobstruction, with up to 25% of these patients showing significant kidney function improvement in a relatively short period, thereby potentially benefiting from NAC. Furthermore, GFR recovery has additional positive clinical implications.

Our study has several limitations. First, its retrospective nature introduces inherent constraints. We could not estimate the duration of the obstruction, and the timing of examinations post-ureteral deobstruction was not pre-established, relying instead on clinician requests. We also lacked data on urine output and were unable to evaluate imaging examinations for all patients, as many did not have initial scans available for analysis. Nevertheless, previous studies have shown that certain imaging parameters, such as parenchymal echogenicity, corticomedullary differentiation, renal perfusion, and parenchymal thickness, do not consistently predict GFR recovery.^14^

This study focused on a specific population of bladder cancer patients and their demographic characteristics, with a particular emphasis on the feasibility of performing urinary diversion to improve GFR recovery and cisplatin eligibility for NAC. By highlighting this issue, we encourage the scientific community to develop tools that can predict when urinary diversion should precede RC to optimize treatment outcomes.

## 5. Conclusion

Some patients with upper tract obstruction secondary to MIBC can benefit from pre-operative urinary diversion, with a mean GFR recovery of 15 mg/mL following deobstruction. Patients with mildly reduced GFR are more likely to become eligible for cisplatin. The time to GFR recovery varies, typically ranging from 1 to 8 weeks.

## Data Availability

The data is available at the institutional database, but cannot be assessed remotely.
